# Clinical utility of sarcoma methylation classifiers in soft tissue sarcoma diagnosis

**DOI:** 10.1007/s00432-026-06494-w

**Published:** 2026-05-16

**Authors:** Justus Osterloh, David E. Reuss, Andreas von Deimling, Hannah Füllgraf, Jannis Heyer, Konrad Kurowski, Clara Kirchhof, Anna Meiertoberens, Ute Lausch, Adrian Schmid, Steffen U. Eisenhardt, David Braig, Alexander Runkel

**Affiliations:** 1 Department of Plastic and Hand Surgery, Faculty of Medicine, Medical Center - University of Freiburg, Freiburg, Germany; 2 Department of Neuropathology, Institute of Pathology, Heidelberg University Hospital, Heidelberg, Germany; 3 Clinical Cooperation Unit Neuropathology, German Cancer Research Center (DKFZ), German Consortium for Translational Cancer Research (DKTK), Heidelberg, Germany; 4 Institute for Surgical Pathology, Faculty of Medicine, Medical Center - University of Freiburg, Freiburg, Germany; 5 Comprehensive Cancer Center Freiburg (CCCF), University Medical Center Freiburg, Freiburg, Germany

**Keywords:** Soft tissue sarcoma, DNA methylation, Tumor classifier, Molecular diagnostics

## Abstract

**Purpose:**

Soft tissue sarcomas are rare malignant tumors of mesenchymal origin characterized by their heterogeneity and diagnosis is challenging despite integrated histopathology and molecular testing. DNA methylation profiling offers an objective approach for tumor classification, but clinical implementation data in soft tissue sarcoma remain limited.

**Methods:**

In this single-center study, 40 tumor samples from 34 patients were evaluated by routine pathology and array-based DNA methylation profiling and classified using two versions of the sarcoma methylation classifier (V12.3 and V13.1).

**Results:**

With version V12.3, 16 samples (40%) achieved a high calibrated score ≥ 0.9, among which 87.5% (14/16) were consistent with the histopathologic diagnosis. With V13.1, 29 samples (72.5%) reached a high calibrated score (≥ 0.9) and the classifier’s diagnosis aligned with the histopathologic diagnosis in 28 cases (96.6%).

**Conclusion:**

Overall, DNA methylation-based classification is a valuable adjunct diagnostic tool to confirm or refine the histopathologic diagnosis. V13.1 substantially improved classification confidence and agreement compared with V12.3. However, a considerable subset of specimens remained below the high-confidence threshold, underscoring the need for further technical and workflow optimization before routine clinical implementation.

## Introduction

Soft tissue sarcomas (STS) are a diverse group of malignant tumors of mesenchymal origin. The 5th edition of the WHO Classification recognizes over 100 STS subtypes, reflecting their broad range of clinical and histopathological features (Choi and Ro 2021). The reported incidence of STS has risen over time, likely due to improved diagnostic techniques and better case documentation (Ressing et al. 2018). However, the standardization of the diagnostic workflow across different centers has proven to be challenging. Despite recent improvements, the diagnosis of STS and subtype classification remains a challenge and still the variability of STS diagnosis among even experienced pathologists is high (Arbiser et al. 2001). For several sarcoma subtypes, there are no distinct molecular markers, and diagnosis is based on morphologic interpretation alone. As a result, novel diagnostic tools are needed to improve classification accuracy.

Epigenetic profiles have been shown to be tissue specific and often contain tumor-specific signatures (Cheng et al. 2023). Accordingly, DNA methylation-based tumor classifiers offer a reliable, objective diagnostic tool to categorize tumors (Capper et al. 2018a; Koelsche and von Deimling 2022). Methylation profiling is gaining more attention in personalized oncology medicine e.g. for diagnosis of CNS tumors. In addition to CNS tumors, changes in the methylomes have also been shown to be entity specific for STS subtypes and analyses may be valuable in the differential diagnosis of histologically indistinguishable entities (Koelsche et al. 2018, 2019; Röhrich et al. 2016; Seki et al. 2015).

Recently, machine learning sarcoma classifier algorithms based on array-generated DNA methylation data were introduced (Koelsche et al. 2021)^,^(Jäger et al. 2025). The initial sarcoma methylation classifier (V12.3) was published in 2021 (Koelsche et al. 2021) and an expanded version (V13.1) was released in 2025 (Jäger et al. 2025). With the update, the reference dataset grew from 1,077 to 4,377 samples and the number of tumor methylation classes increased from 62 to 89 (Jäger et al. 2025). Both classifiers use a Random Forest algorithm and are publicly available. Beyond classifying tumors into established subgroups, they can also discover novel subgroups (Jäger et al. 2025; Noushmehr et al. 2010; Reinhardt et al. 2018; Sturm et al. 2012). However, unlike in CNS tumors, clinical data on DNA methylation-based classification in sarcomas are still limited. Therefore, we aimed to evaluate the clinical utility of the sarcoma methylation classifiers in practice and to identify their potential limitations.

## Materials and methods

For this study, we prospectively collected samples from patients with soft tissue sarcomas treated at our center from January 2023 to September 2024 using the study protocol described by Runkel et al (Runkel et al. 2023). The samples were collected either as pretherapeutic core-needle biopsies (CNB) or after resection. Some samples were taken after neoadjuvant radiotherapy (NRT), while others were from non-irradiated tissue. Samples were either formalin-fixed, paraffin-embedded (FFPE) or fresh frozen. Genomic DNA was extracted using the DNeasy Blood & Tissue Kit (Qiagen GmbH, Hilden, Germany; Cat. No. 69504) according to the manufacturer’s instructions. DNA concentration and purity were quantified using the Qubit 1× dsDNA High Sensitivity Assay Kit (Thermo Fisher Scientific Inc., Waltham, MA, USA; Cat. No. Q33231). The Illumina Infinium HumanMethylation450 (450k) array was used to obtain genome-wide DNA methylation data for tumor according to the manufacturer’s instructions (Illumina, San Diego, USA). DNA methylation signals were analyzed using the R/Bioconductor package minfi (version 1.4.0), following previously described methods (Jäger et al. 2025; Koelsche et al. 2021; Lyskjaer et al. 2021). All samples were categorized into subtypes using versions V12.3 and V13.1. In line with previous publications a calibrated score ≥ 0.9 indicated successful classification (Jäger et al. 2025; Koelsche et al. 2021). Histopathological reports including immunohistochemistry and molecular analyses were performed by two specialist sarcoma pathologists. In selected cases requiring reference pathology, samples were consistently reviewed at the same external sarcoma reference center. These diagnoses served as the reference standard for clinical decision-making and were compared with the results of the DNA methylation classifier.

Medical records were searched for clinical data and histopathological and molecular pathological analysis and diagnosis. Ethical approval was obtained from the Ethics Committee of the Albert-Ludwig-University Freiburg, Germany (study number EK 21-1735). The study was conducted in accordance with the Declaration of Helsinki. All statistical analyses were conducted using RStudio (version 2024.09.0 + 375; R Foundation for Statistical Computing, Vienna, Austria). McNemar’s test assessed improvements in paired classifications, while Chi-square test evaluated changes in score distribution and Wilcoxon signed-rank evaluated changes in classifier confidence. A significance level of *p* < 0.05 was applied.

## Results

Between January 2023 and September 2024, 40 samples were collected from 34 patients with a histopathologic diagnosis of soft tissue sarcoma, including 24 samples from male and 16 from female patients. Sixteen samples (40%) were obtained by core-needle biopsy and 24 (60%) from surgical resections. Tissue preservation included 27 fresh frozen (67.5%) and 13 FFPE (32.5%) samples. Twenty-four specimens (60%) were collected after neoadjuvant radiotherapy, and 16 (40%) from non-irradiated tissue (Table 1).

**Table 1:** Patient and Sample Characteristics (*n* = 40)

Patient/Sample Characteristics		Values/Numbers	%
Sex	Male	24	60
	Female	16	40
Post neoadjuvant radiation	Yes	24	60
	No	16	40
Tissue	Fresh frozen	27	67.5
	FFPE	13	32.5
Samples	CNB	16	40
	Resection specimen	24	60
Histopathological subtypes	US (NOS + UPS)	21	52.5
	MFS	11	27.5
	PLS	3	7.5
	LMS	2	5
	MLS	2	5
	AS	1	2.5

After histopathological and molecular analysis, all included samples were additionally analyzed with both versions of the sarcoma methylation classifier. The most common histopathologic diagnosis was Sarcoma not otherwise specified (NOS, 11/40) and Undifferentiated Pleomorphic Sarcoma (UPS, 10/40) grouped together as Undifferentiated Sarcoma (US, 21/40), followed by Myxofibrosarcoma (MFS, 11/40). Other histopathologic subtypes included Pleomorphic Liposarcoma (PLS, 3/40), Leiomyosarcoma (LMS, 2/40), Myxoid Liposarcoma (MLS, 2/40) and Angiosarcoma (AS, 1/40).

**Fig. 1:**
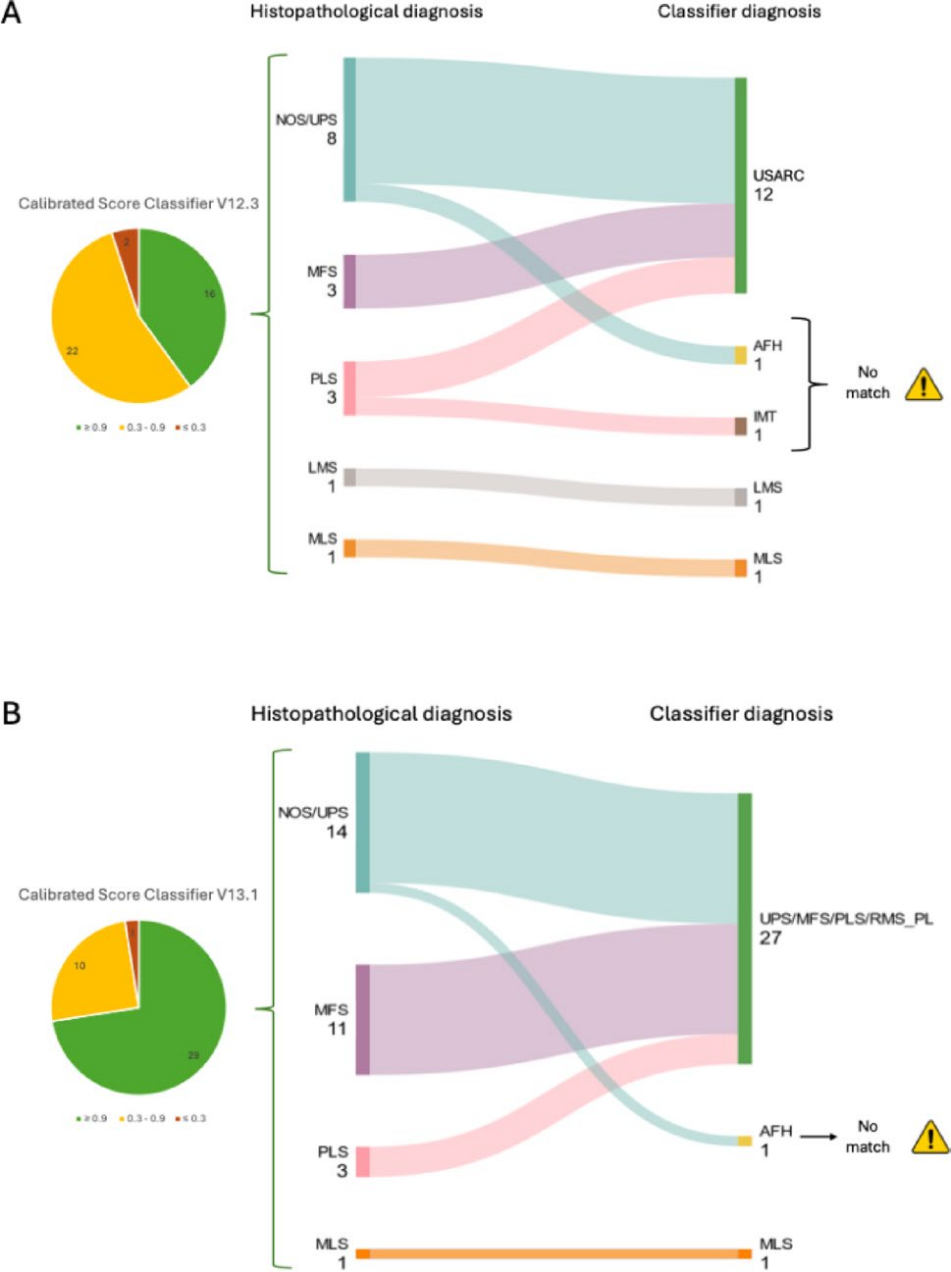
Calibrated score of versions V12.3 (A) and V13.1 (B) of the sarcoma methylation classifier. Sankey Plot of all samples with a high calibrated score ≥ 0.9. The classifier V12.3 combines entities UPS/MFS/PLS and V13.1 combines UPS/MFS/PLS/RMS as undifferentiated sarcoma. The mismatches are marked as “no match”. The classifier V12.3 indicated a non-malignant or low-malignant diagnosis for 2 samples that were originally diagnosed as malignant sarcomas, while classifier V13.1 suggested a non-malignant diagnosis for 1 sample initially classified as malignant

Initially, version V12.3 of the sarcoma methylation classifier was used, and a clear match to a reference group was found in 16 samples (16/40, 40%), with a calibrated score of ≥ 0.9. Of the samples with a high calibrated score, the diagnosis was in concordance with the histopathologic diagnosis in 87.5% (14/16) (Fig. 1). However, in two cases where the classifier produced a high calibrated score despite a mismatch, the subsequent diagnosis was non-malignant or low malignant. These two samples were initially diagnosed as NOS or PLS by the pathologists, but the classifier classified them as Angiomatoid Fibrous Histiocytoma (AFH) and Inflammatory Myofibroblastic Tumor (IMT) (Fig. 1). Of the samples with a calibrated score lower than 0.9, the prediction of the classifier agreed with the histopathological diagnosis in only 8 cases (8/24, 33.3%).

The latest version of the sarcoma methylation classifier (version V13.1) achieved a high-calibrated score of ≥ 0.9 in 29 samples (29/40, 72.5%), with the classifier’s prediction aligning with the histopathological diagnosis in all but one case (28/29, 96.6%). In the single discordant case, the classifier predicted a low-grade malignant diagnosis (AFH), whereas the histopathological analysis diagnosed a high-grade undifferentiated pleomorphic sarcoma (UPS). When the calibrated score of the latest version of the classifier was < 0.9, the prediction was in line with the histopathological diagnosis in 5 of 11 cases (45.5%).

**Fig. 2:**
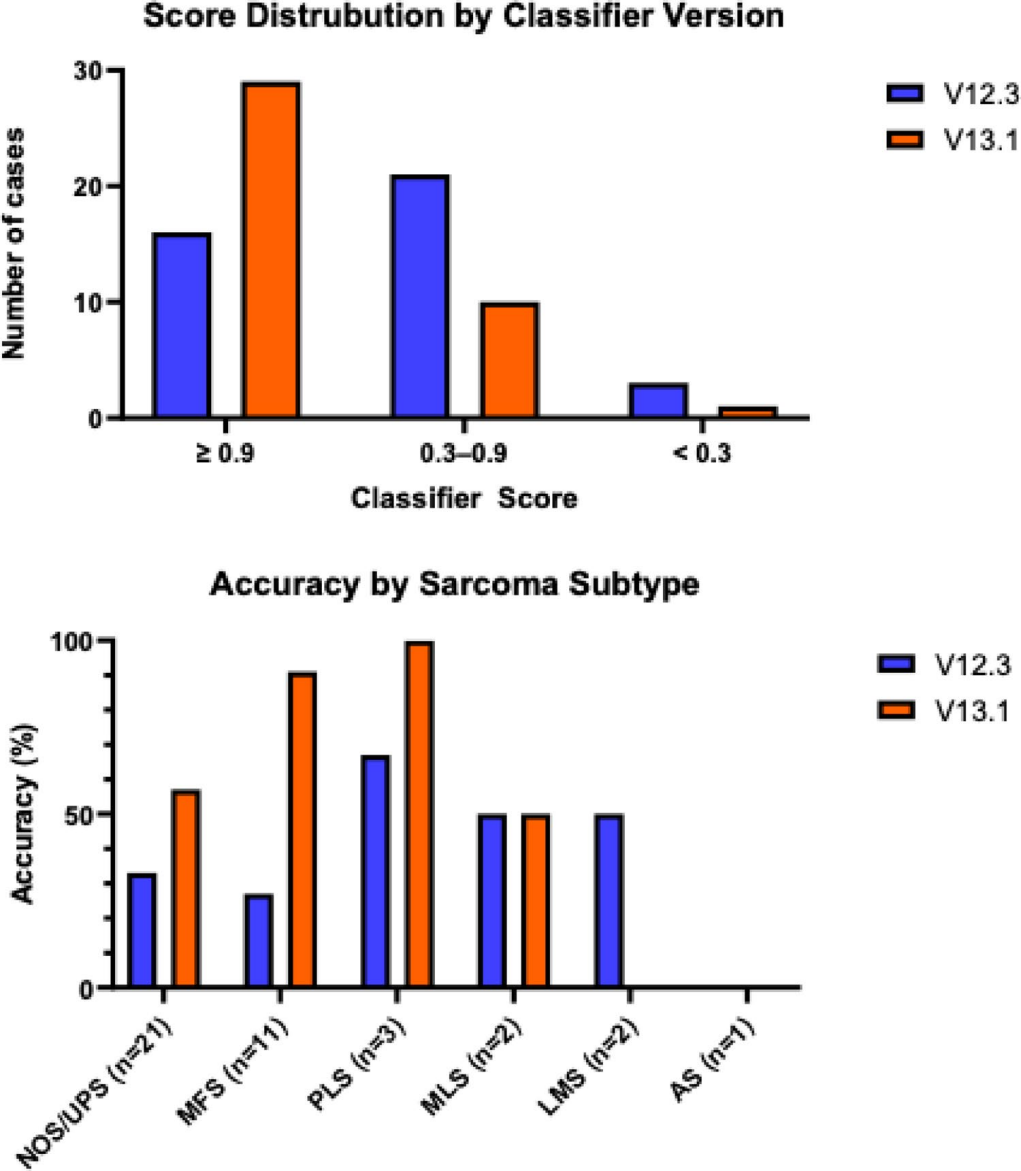
Score distribution and accuracy comparison by subtype of versions V12.3 and V13.1 of the sarcoma methylation classifier. (A) Calibrated score distribution of the two classifier versions, categorized into three confidence ranges: >0.9, 0.3–0.9, and < 0.3, illustrating shifts in classification confidence. (B) Accuracy (%) of both classifier versions across different sarcoma subtypes, highlighting performance variations by histological category

V13.1 significantly outperformed V12.3 across all conditions (*p* < 0.001, Cohen’s d = 0.68) (Table 2). V13.1 showed higher accuracy (median scores closer to 1.0) and substantially lower variability, particularly for fresh-frozen (native) tissue samples (a 97% reduction in MAD) (Table 2; Fig. 2). Neither prior radiotherapy nor tissue type (FFPE vs. native) significantly affected classifier performance (*p* > 0.05 for all comparisons), indicating robust performance across different clinical settings (Supplementary Tables 1–4). Notably, V13.1 virtually eliminated the performance gap between FFPE and fresh tissue (median scores 0.9781 vs. 0.9971 in V13.1, compared to 0.6708 vs. 0.8678 in V12.3). V13.1 also exhibited a more pronounced negative skewness (scores clustering near the maximum).

**Table 2:** Statistical analysis of the performance (calibrated score) of the Sarcoma Methylation Classifier version V12.3 vs. version V13.1

Overall (*n* = 40)	V12.3	V13.1
Mean	0.7254	0.9215
Median	0.8425	0.9961
Minimum	0.1310	0.2386
Maximum	0.9999	1
Q1 (25th percentile)	0.5340	0.8940
Q3 (75th percentile)	0.9717	0.9999
MAD	0.1539	0.0039
Skewness	-0.7476	-3.0312
Test	**Statistic**	***p*** **-value**
Wilcoxon Signed-Rank Test	W = 7.00	*p* < 0.001
Paired t-Test	t = 4.2585 (df = 39)	*p* < 0.001
Effect Size: Percent Improved in V13.1	82.5%	-

## Discussion

### Diagnostic challenges in sarcoma management

Sarcomas are rare malignant tumors of mesenchymal origin, accounting for less than 1% of adult cancers but a higher proportion up to 14% of pediatric malignancies (Clark et al. 2005; HaDuong et al. 2015). One major challenge in sarcoma diagnosis is their morphological and genetic heterogeneity, which complicates accurate classification and treatment selection. Traditional diagnostic approaches rely on histopathology, immunohistochemistry (IHC), and increasingly molecular genetics (e.g. fluorescence in situ hybridization (FISH), RNA fusion testing and next-generation sequencing (NGS). While these methods provide valuable information, they are not always definitive, particularly for sarcomas with ambiguous morphological features or overlapping molecular alterations. Misdiagnosis can lead to inappropriate treatment with significant impacts on patient outcomes (Jain et al. 2010). DNA methylation profiling has emerged as a promising tool for improving sarcoma classification. The sarcoma methylation classifier leverages genome-wide DNA methylation patterns to classify sarcomas with high specificity and sensitivity (Koelsche et al. 2021). It is based on machine learning algorithms and is trained on extensive methylation datasets.

To date, DNA methylation-based classifiers are used in the clinical practice for classification of central nervous system (CNS) tumors (Capper et al. 2018a, b; Djirackor et al. 2021; Karimi et al. 2019). Analyzing and identifying specific methylation patterns of CNS tumors lead to the identification of novel subtype classifications with clinical relevance (Reinhardt et al. 2018; Sturm et al. 2012). Furthermore, integrating the CNS classifier into clinical practice changed diagnoses and treatments, contributing to improved outcomes in patients with CNS tumors (Karimi et al. 2019). The integration of DNA methylation-based analyses is now expanding to other oncology fields. Beyond CNS tumors, analysis of epigenetic patterns is being explored for other malignancies including cancer of unknown primary (CUP), melanoma, and lymphoid malignancies (Duran-Ferrer et al. 2020; Hao et al. 2017; Micevic et al. 2017; Moran et al. 2016; Sill et al. 2020). In this single-center study, we describe our experience with the DNA methylation-based tumor classifier for soft tissue sarcomas from a highly heterogeneous patient population, with samples from different clinical settings and diagnostic stages.

## Performance of DNA methylation classifiers and technical considerations

In the initial publication introducing the sarcoma methylation classifier, 75% of samples reached a high calibrated score ≥ 0.9, and within this subset 82% of classifier predictions were concordant with the initial histopathologic diagnosis (Koelsche et al. 2021). In the validation study by Lyskjær et al., only 55% of the analyzed samples reached a calibrated score ≥ 0.9, and within this group, 83% of classifier predictions were concordant with the initial diagnosis (Lyskjaer et al. 2021). In our analysis, with the initial classifier version V12.3, only 40% of samples achieved a high calibrated score, and in 87.5% of these cases the classifier result was concordant with the histopathologic analysis. The samples in our study were highly heterogeneous, as they included both pre- and post-radiation specimens as well as FFPE and native tissue samples. Importantly, we did not observe significant performance differences between classifier versions under these varying conditions. These findings align with current literature and highlight a key advantage of the classifier: its relatively low sample requirements for analysis (Roohani et al. 2022).

However, a major limitation of our study is the lack of systematic assessment of tumor cell content. In contrast, in the validation study of the classifier by Lyskjaer et al., only samples with a tumor content of at least 40% were analyzed (Lyskjaer et al. 2021), and in the initial publication by Koelsche et al., a minimum tumor cell content of 70% is recommended (Koelsche et al. 2021). Insufficient tumor cell representation, particularly in post-radiation or inflammatory specimens, may dilute the tumor-specific methylation signal and contribute to lower confidence scores or divergent classifications.

Furthermore, the pretherapeutic biopsy samples in our study were collected ultrasound-guided. To increase tumor cell content and the rate of high-calibrated scores in future analysis, improved sampling techniques could target the viable tumor region more precisely. In prostate cancer, for example, MRI/ultrasound-fusion guided biopsies are used to improve sampling efficiency. This approach detects more clinically significant tumors with fewer cores compared to standard ultrasound-guided biopsies (Siddiqui et al. 2013; Valerio et al. 2015). Hypothetically, similar strategies could be applied to sarcoma diagnostics to ensure representative biopsy samples and potentially improve classifier reliability.

Careful evaluation of the tissue selected for methylation analysis is mandatory and can impact classifier performance and the final diagnosis. This is particularly relevant in cases where the classifier yields a high calibrated score but suggests a benign or low-grade diagnosis, whereas the histopathology indicates a high-grade tumor. In a clinical scenario where the classifier’s suggestion prompts a referential pathology review or even a change in diagnosis, this approach could potentially avoid unnecessary radical treatment strategies and their associated risks and side effects, positively impacting overall patient outcomes and quality of life.

In our cohort, in the one case where both classifier versions predicted a low-grade diagnosis (AFH) but histopathology indicated a high-grade tumor, the patient developed pulmonary metastasis only 3 months after resection. This outcome suggests the tumor was indeed high-grade despite the classifier result, underscoring that such results must be interpreted with caution. A plausible explanation for the classifier’s divergent classification could be a high proportion of infiltrating non-tumor cells in the sample, which may have affected the methylation profile.

In our cohort, two samples analyzed with version V12.3 and one sample with version V13.1 were classified as IMT or AFH despite histopathologic diagnoses of high-grade sarcoma. All these samples originated from irradiated tumors, suggesting that therapy-induced changes, prominent inflammatory background and reduced tumor cell content may have contributed to the divergent classifications. Notably, Miettinen et al. also reported that the classifier performs inconsistently for IMTs, often producing low-confidence results or divergent classifications. This underscores that both sample composition and intrinsic biological features of certain mesenchymal entities, such as IMT, can limit classifier accuracy (Miettinen et al. 2024).

With version V13.1 of the sarcoma methylation classifier, we observed significant improvements in score distribution and subtype classification accuracy (Fig. 2). Differences between classifier versions may partly reflect updates to the underlying reference cohort and class definitions as described in the respective classifier publications (Jäger et al. 2025; Koelsche et al. 2021). Furthermore, the consolidation of several high-grade sarcoma entities, including UPS, MFS, PLS, and pleomorphic rhabdomyosarcoma (P-RMS), into a single classification group in version V13.1 enhances classification robustness but reduces diagnostic specificity. This aggregation likely reflects genuine biological and epigenetic similarities among these pleomorphic sarcomas rather than a technical limitation of the classifier(Carvalho et al. 2019; Hornick 2018).

In our cohort, 27 out of 29 high-score samples were assigned to this pooled category. Clinically, this loss of subclassification has limited therapeutic impact at present, as currently guidelines recommend a uniform therapeutic approach for most high-grade sarcomas. However, exceptions already exist and few subtypes have established, subtype-adapted regimens or distinct sensitivities (e.g., rhabdomyosarcoma with multi-agent protocols (Bisogno et al. 2019) or myxoid liposarcoma with marked radiosensitivity and activity of trabectedin(Sanfilippo et al. 2023). As knowledge of sarcoma subtypes and their differential responses to radiotherapy and chemotherapy expands, more precise subclassification may become clinically significant for guiding personalized treatment strategies.

In addition, technical aspects related to array platform compatibility should be considered. Version V12.3 of the sarcoma classifier was originally developed using data generated on the Illumina 450k and EPIC v1 platforms (Koelsche et al. 2021). Classifier version V13.1 also integrates the recently introduced Infinium MethylationEPIC v2.0 array (Jäger et al. 2025), which provides increased CpG coverage. However, cross-platform analyses are usually standardized by restricting them to the set of probes that are common to both platforms. Consequently, the additional CpG sites present on newer array versions may not be fully utilized for classification purposes (Dermawan 2025). Furthermore, differences in probe composition between array generations may limit comparability with existing reference datasets.

## Clinical integration

**Fig. 3:**
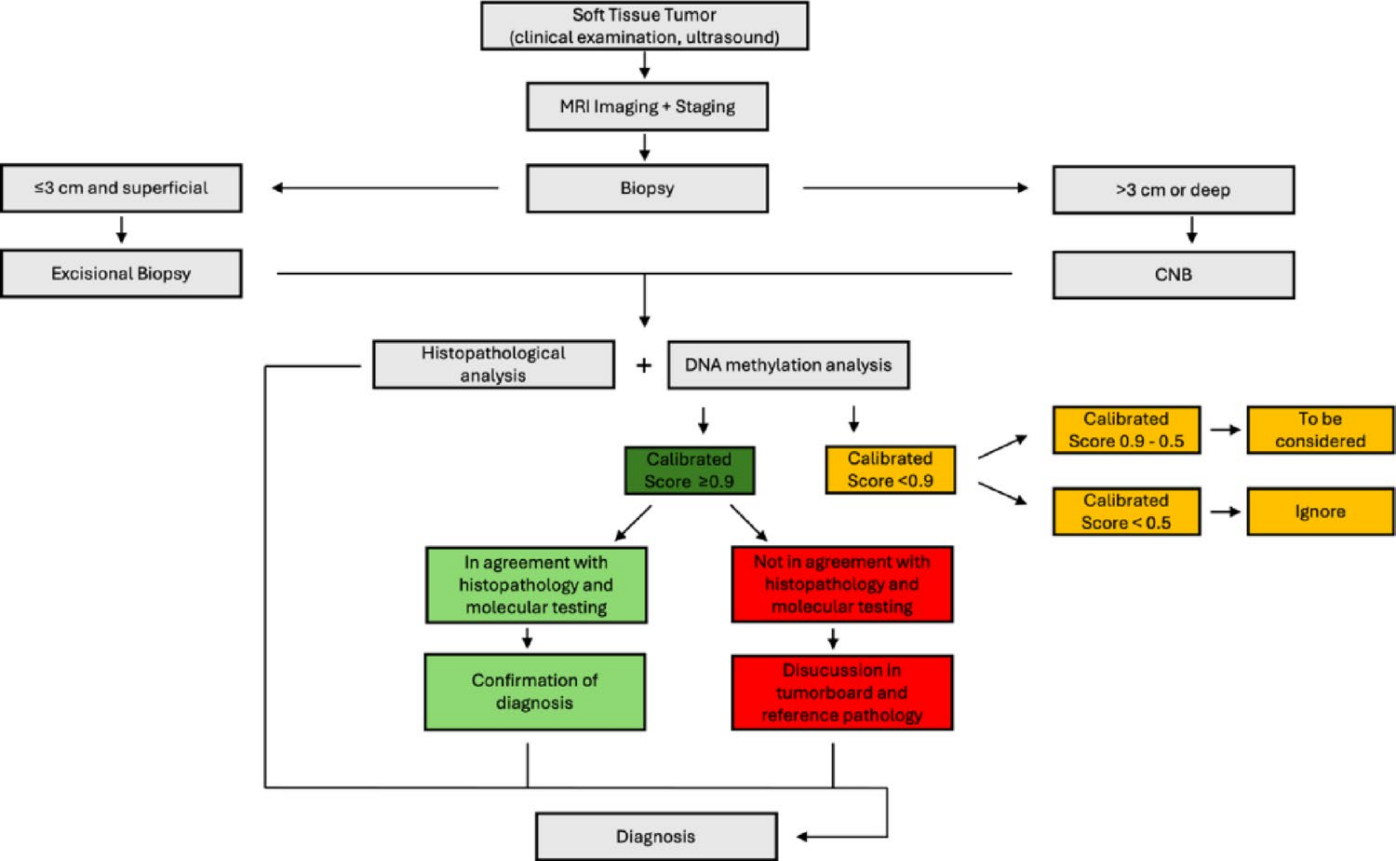
Proposed diagnostic algorithm for the clinical implementation of the DNA methylation classifier for selected cases of soft tissue sarcomas as an additional diagnostic tool

While DNA methylation-based classification is highly precise, it should not be used in isolation. Traditional histopathology, immunohistochemistry, and genetic testing remain crucial for accurate diagnosis. A purely epigenetic approach might overlook key histological and clinical features, potentially leading to misdiagnosis if not carefully interpreted. Pathologists and clinicians must integrate methylation data with other diagnostic tools rather than using it as a standalone method. To optimize clinical application of the sarcoma DNA methylation classifier, we propose a diagnostic algorithm (Fig. 3).

We emphasize that DNA methylation profiling should be applied in selected cases following thorough clinical, histopathological and molecular evaluation. A calibrated score ≥ 0.9 indicates a high-confidence classification, however, all classifier results must be interpreted within an integrated clinicopathological framework. Calibrated scores < 0.9 reflect reduced confidence and should be interpreted with increasing caution as the score decreases. Very low scores (< 0.5) provide only weak diagnostic value and should not be considered supportive evidence.

All cases should be discussed in an interdisciplinary tumor board, and if discrepancies remain unresolved, referral for expert pathology review is recommended.

Beyond its use as an adjunct in diagnostically challenging cases, DNA methylation profiling may also hold promise for the primary diagnostic workup of soft tissue tumors, including those with uncertain or potentially benign morphology. Current classifiers, however, have been trained mainly on malignant entities and therefore show limited sensitivity for benign or borderline lesions, which are often assigned low-confidence results(Henzinger et al. 2024; Miettinen et al. 2024). Expanding the reference dataset to include benign and intermediate tumor methylomes could substantially enhance the diagnostic reach of this approach, enabling earlier and more reliable distinction between benign, intermediate, and malignant lesions in routine pathology.

Despite its diagnostic potential, the integration of DNA methylation profiling into routine clinical practice is associated with economic and logistical considerations. While per-sample costs of array-based methylation profiling are lower than those of comprehensive sequencing approaches such as whole-genome sequencing (WGS), they remain substantial and are not yet supported by formal cost-effectiveness analyses in sarcoma (Peters et al. 2024; Pidsley et al. 2016). From a logistical perspective, testing is currently limited to specialized centers, often requiring inter-institutional sample transfer, which may cause additional complexity. Laboratory processing time is approximately four days. However, real-world turnaround times are typically closer to one to two weeks. Methylation profiling can be performed in parallel with conventional diagnostics, although it is frequently applied as a second-line test in diagnostically unresolved cases. A key advantage of this approach is the generation of multiple layers of diagnostic information, including tumor classification, copy number profiles (CNV), and MGMT promoter methylation status within a single assay, potentially reducing the need for sequential ancillary testing (Koelsche et al. 2021). Long-read sequencing (LRS) technologies may enable simultaneous detection of gene fusions, structural variants, copy number alterations, and methylation patterns within a single assay, but remain investigational in sarcoma diagnostics (Ermini and Driguez 2024; Iluz et al. 2023). However, for genetically simple sarcoma subtypes, targeted assays such as FISH or RT-PCR remain more rapid and cost-efficient. While robust implementation data are available for central nervous system tumors (White et al. 2023), comparable evidence for sarcomas remains limited, and current classifiers have not yet reached the same level of maturity.

## Conclusion

The sarcoma methylation classifier is a powerful tool that can enhance diagnostic accuracy and provide valuable epigenetic insights. Methylomic profiling has the potential to refine diagnostic classification and may contribute to improved stratification in selected clinical scenarios. However, clinical translation requires standardized methodologies, subtype-specific trials, and validation in prospective cohorts. Integrating methylation data with genomic and transcriptomic analyses will be critical for realizing precision oncology in STS.

**Tables**.

## Data Availability

The DNA methylation data (IDAT files) generated in this study are being deposited in the Gene Expression Omnibus (GEO).
